# Effects of sleep disturbances and circadian rhythms modifications on cognition in breast cancer women before and after adjuvant chemotherapy: the ICANSLEEP-1 protocol

**DOI:** 10.1186/s12885-023-11664-x

**Published:** 2023-12-01

**Authors:** Clara Elia, Laura de Girolamo, Bénédicte Clarisse, Melvin Galin, Stéphane Rehel, Patrice Clochon, Franck Doidy, Shailendra Segobin, Fausto Viader, Mikaël Naveau, Nicolas Delcroix, Carine Segura-Djezzar, Jean-Michel Grellard, Justine Lequesne, Olivier Etard, Tristan Martin, Gaëlle Quarck, Francis Eustache, Florence Joly, Bénédicte Giffard, Joy Perrier

**Affiliations:** 1 Normandie Univ, UNICAEN, PSL Université, EPHE, INSERM, U1077, CHU de Caen, GIP Cyceron, Neuropsychologie et Imagerie de la Mémoire Humaine, Caen, 14000 France; 2 Clinical Research Department, Centre François Baclesse, Caen, 14076 France; 3 Cancer and Cognition Platform, Ligue Nationale Contre le Cancer, Caen, 14076 France; 4 Medical Oncology Department, Centre François Baclesse, Caen, 14076 France; 5 Neurology Department, CHU de Caen, Caen, 14000 France; 6 Pôle des Formations et de Recherche en Santé, 2 rue des Rochambelles, Caen Cedex, CS-14032 France; 7 ANTICIPE (Interdisciplinary Research Unit for the Prevention and Treatment of Cancer), INSERM Unit 1086, Caen, France; 8 Faculty of Sciences and Technologies, Le Mans University, Avenue Olivier Messiaen, Movement, Interactions, Performance, Le Mans, 4334, 72000 MIP, EA France; 9 Normandie Université, UNICAEN, INSERM, COMETE U1075, CYCERON, CHU Caen, Caen, 14000 France; 10 Normandie Université, UNICAEN, CNRS UAR 3408, INSERM US-50, GIP Cyceron, Caen, France

**Keywords:** Breast cancer, Adjuvant chemotherapy, Sleep structure, Circadian rhythms, Cognition, White matter integrity

## Abstract

**Background:**

Many patients treated for breast cancer (BC) complain about cognitive difficulties affecting their daily lives. Recently, sleep disturbances and circadian rhythm disruptions have been brought to the fore as potential contributors to cognitive difficulties in patients with BC. Yet, studies on these factors as well as their neural correlates are scarce. The purpose of the ICANSLEEP-1 (Impact of SLEEP disturbances in CANcer) study is to characterize sleep using polysomnography and its relationship with the evolution of cognitive functioning at both the behavioral and the neuroanatomical levels across treatment in BC patients treated or not with adjuvant chemotherapy.

**Methods:**

ICANSLEEP-1 is a longitudinal study including BC patients treated with adjuvant chemotherapy (n = 25) or not treated with adjuvant chemotherapy (n = 25) and healthy controls with no history of BC (n = 25) matched for age (45–65 years old) and education level. The evaluations will take place within 6 weeks after inclusion, before the initiation of chemotherapy (for BC patients who are candidates for chemotherapy) or before the first fraction of radiotherapy (for BC patients with no indication for chemotherapy) and 6 months later (corresponding to 2 weeks after the end of chemotherapy). Episodic memory, executive functions, psychological factors, and quality of life will be assessed with validated neuropsychological tests and self-questionnaires. Sleep quantity and quality will be assessed with polysomnography and circadian rhythms with both actigraphy and saliva cortisol. Grey and white matter volumes, as well as white matter microstructural integrity, will be compared across time between patients and controls and will serve to further investigate the relationship between sleep disturbances and cognitive decline.

**Discussion:**

Our results will help patients and clinicians to better understand sleep disturbances in BC and their relationship with cognitive functioning across treatment. This will aid the identification of more appropriate sleep therapeutic approaches adapted to BC patients. Improving sleep in BC would eventually help limit cognitive deficits and thus improve quality of life during and after treatments.

**Trial registration:**

NCT05414357, registered June 10, 2022.

**Protocol version:**

Version 1.2 dated March 23, 2022.

## Background

Breast cancer (BC) is the most frequent cancer in women, with 2.26 million cases worldwide in 2020 [1, 2]. Cognitive complaints reported by BC patients [3, 4] have attracted the interest of many researchers, making BC a standard model for studying cognitive decline in non-central nervous system (non-CNS) cancers [5, 6]. These complaints are often reported during and after adjuvant chemotherapy but can also be found before treatment in 20–30% of BC patients [7, 8]. The majority of them refer to memory loss, slowed processing speed, and executive dysfunction [6, 9]. Although subtle in nature, these cognitive difficulties negatively affect the quality of life of BC patients [10, 11] and may persist up to 20 years after chemotherapy [12]. In addition to the effects of treatments, sleep disturbances and circadian rhythm disruptions have been recently brought to the fore as potential contributors to cognitive difficulties in BC patients [13–16]. Sleep is known to play a crucial role in maintaining cognitive functioning, such as episodic memory [17] and executive functioning [18]. However, the association between sleep and cognitive decline in BC still remains largely unknown [14] and thus requires further investigations.

Complaints about troubled sleep before, during, and after chemotherapy are frequent in BC patients, with symptoms of insomnia being the most significant complaint [19, 20]. Moreover, sleep disturbances may be the result of circadian rhythm disruptions that have been highlighted elsewhere in BC patients. Indeed, sleep behavior depends on the interaction of a homeostatic sleep drive with endogenous circadian rhythms [21]. As a side note, both sleep and circadian rhythm disruption have been highlighted before cancer diagnosis and could be predisposing factors for BC incidence, highlighting the need to further identify and address such alterations in BC patients [22–24].

Previous reports have shown alterations of circadian rhythms following chemotherapy, particularly reflected by salivary cortisol profiles and rest-activity rhythms, which are easy to measure and provide reliable information about circadian rhythms. Cortisol is the primary product of the hypothalamic-pituitary-adrenal (HPA) axis [25], and previous reports have shown flattened diurnal cortisol patterns in BC patients following chemotherapy [26, 27]. Moreover, previous studies have shown dysregulation of the rest-activity rhythm using actigraphy in BC patients treated with chemotherapy [28–30]. The results of these studies have shown a lower amplitude of activity levels (difference between the maximum and minimum of the best fitting curve) as well as a lower mesor (the rhythm-adjusted mean of the best fitting curve).

Due to its ease of use, many studies have used actigraphy to assess sleep in BC patients before or during chemotherapy. Some showed a shortened sleep time and more frequent awakenings during chemotherapy compared to the status before the beginning of the treatment [31, 32], whereas others showed no significant difference in terms of frequent awakenings and lower sleep quality before and during chemotherapy [33]. Before chemotherapy, Ancoli-Israel and colleagues [34] showed lower sleep efficiency ([current sleep time/time in bed] × 100) in BC patients while Berger and colleagues [35] showed better sleep efficiency compared to previously established norms. Meanwhile, polysomnography (PSG), the gold-standard method to measure sleep quality and quantity, is rarely used in cancer research [15, 19, 36]. For instance, Silberfab and colleagues [37] did not find any significant difference in sleep patterns between BC patients after chemotherapy and age-matched healthy women. Two studies found sleep structure modifications in BC patients after chemotherapy, notably lower sleep efficiency and higher sleep onset latency [38, 39]. Roscoe and colleagues [36] noted that BC patients slept for longer at the end of chemotherapy than before the start of chemotherapy. Several methodological limitations may have led to these discrepant findings. Although these studies have provided informative results, their main limitation is the lack of control groups. Having a control group of subjects with no history of cancer and even a control group of BC patients without chemotherapy treatment would allow for determining whether sleep modifications are related to cancer per se and/or to chemotherapy. Moreover, the heterogeneity or sample sizes of the groups studied may have also limited the statistical power of the results. New studies are thus needed to further knowledge about sleep modifications in BC patients before and after chemotherapy.

Both sleep quantity and quality are known to contribute to cognitive functioning in both young and old healthy volunteers [18, 40]. Besides characterizing sleep structure, PSG also provides objective quantitative measurements of memory consolidation during sleep through the quantification of sleep spindles (transient 12–15 Hz oscillations generated within thalamocortical loops) and slow waves (cortical < 1 Hz oscillations). Memory consolidation is an active process that integrates newly encoded information into long-term memory networks and is more efficient during sleep [41]. As an example, a reduction in sleep spindle density has been associated with lower memory consolidation in older adults [42–44]. Currently, this topic is rarely addressed in BC patients: only one study has reported a higher spindle frequency and lower slow wave amplitude in BC patients not treated with chemotherapy compared to age-matched healthy women [45]. Given that the link between sleep and cognitive functioning has mostly been assessed using subjective scales and self-report questionnaires in BC [14], new data are required to clarify the relationship between sleep modifications and cognitive deficits in BC patients, both before and after chemotherapy. Moreover, previous reports have shown both white and grey matter alterations in BC patients before and after chemotherapy [46–48]. Given the role of white matter integrity in spindle propagation, as well as the contribution of both white and grey matter alterations in cognitive difficulties in BC patients, one may expect that alterations in grey and white matter could influence both memory consolidation during sleep and cognitive functioning. It is therefore essential to quantify the predictive role of grey and white matter modifications in sleep and its relationship with cognitive functioning. Additionally, some studies have found grey matter reductions among BC patients during and after chemotherapy [49–51] and others have related it to worse neuropsychological performance compared to healthy control groups [52, 53].

In the ICANSLEEP-1 study herein presented, we aim to prospectively assess sleep modifications through PSG among BC patients initiating chemotherapy, as compared to healthy age-matched women and BC patients with no indication to receive chemotherapy.

## Methods

The ICANSLEEP-1 study is a single-center longitudinal study where sleep will be prospectively assessed using PSG among three groups: BC patients treated with chemotherapy, BC patients without chemotherapy, and cancer-free women. Self-report questionnaires and neuropsychological tests will be used to assess participants’ complaints and quality of life as well as their cognitive profiles. Rest-activity and cortisol rhythms will be assessed using actigraphy and saliva recollection, respectively. Polysomnography will serve to quantify sleep structure, that is, sleep quantity and quality. Neuropsychological tests will evaluate participants’ cognitive functioning. Structural magnetic resonance imaging (MRI) will be used to quantify both grey and white matter integrities, respectively. The ICANSLEEP-1 protocol and this manuscript have been written in accordance with standard protocol items, following recommendations for interventional trials (SPIRIT).

### Study objectives

The main objective is to longitudinally quantify sleep modifications associated with BC and chemotherapy using PSG.

The secondary objectives are to:

- Assess the relationship between the presence of sleep disturbances and poor cognitive performance, both before and after chemotherapy.
- Quantify the predictive role of white matter integrity alterations in explaining such a relationship.
- Determine whether the presence of sleep disturbances is associated with circadian disruptions in BC patients both before and after chemotherapy.

### Participants

Three groups of women aged between 45 and 65 years will be recruited: 25 BC patients scheduled to receive adjuvant chemotherapy (CHE), 25 BC patients treated with radiotherapy alone with no indication of chemotherapy (NCH), and 25 healthy female controls (CTL). BC patients in the NCH group will be matched for age and level of education to BC patients from the CHE group. Similarly, the female volunteers enrolled in the CTL group will be matched to BC patients for age and level of education. Both pre- and postmenopausal women will be included, and information regarding menopausal status as well as the date of the beginning of menopause will be collected for all participants.

Eligible BC patients will be asked to participate in the ICANSLEEP-1 study by the medical and/or radiation oncologists. An explanation of the study and an information note will be given to them. Eligibility criteria are indicated in Table 1. Patients will be enrolled in the study once they provide their written informed consent.

**Table 1:** ICANSLEEP-1 inclusion and non-inclusion criteria

	Inclusion Criteria	Exclusion Criteria
Breast cancer patients and Healthy individuals	Age between 45–65 yo French native speakers At least on level 3 (end of primary schools) of the Barbizet scale Insomnia Sleep Index (ISI > 7) Women who don’t work on night shifts Women who signed the consent form to participate in the study	Neurological sequelae Personality disorders and progressive psychiatric disorder Drug use and/or heavy drinking Women with contraindication to MRI Women with uncorrected vision problem Women with treated sleep apnea syndrome Women with a treatment that hasn’t been stabilized for at least 3 months (hypnotics, antidepressants, anxiolytics)
Healthy individuals	Women with no history of cancer	-
Breast cancer patients	Operated for local breast cancer Treated with adjuvant Chemotherapy +/- Radiotherapy Absence of pre-existing cognitive impairment at cancer diagnosis	Metastatic cancer

Healthy volunteers will be recruited from the general population through associations, an advertisement in Centre François Baclesse, or by acquaintance, for example. Eligible women will be asked to participate by the physicians from our laboratory and will receive an information file. Women who meet all eligibility criteria (Table 1) will be asked to sign the consent form to be enrolled in the study.

### Study sites

The study will be conducted in the comprehensive cancer center François Baclesse (Caen, France) for BC patients and in our laboratory at Normandie University (Caen, France) for healthy women, as indicated on https://clinicaltrials.gov/ct2/show/NCT05414357.

### Modalities of participation

**Fig. 1:**
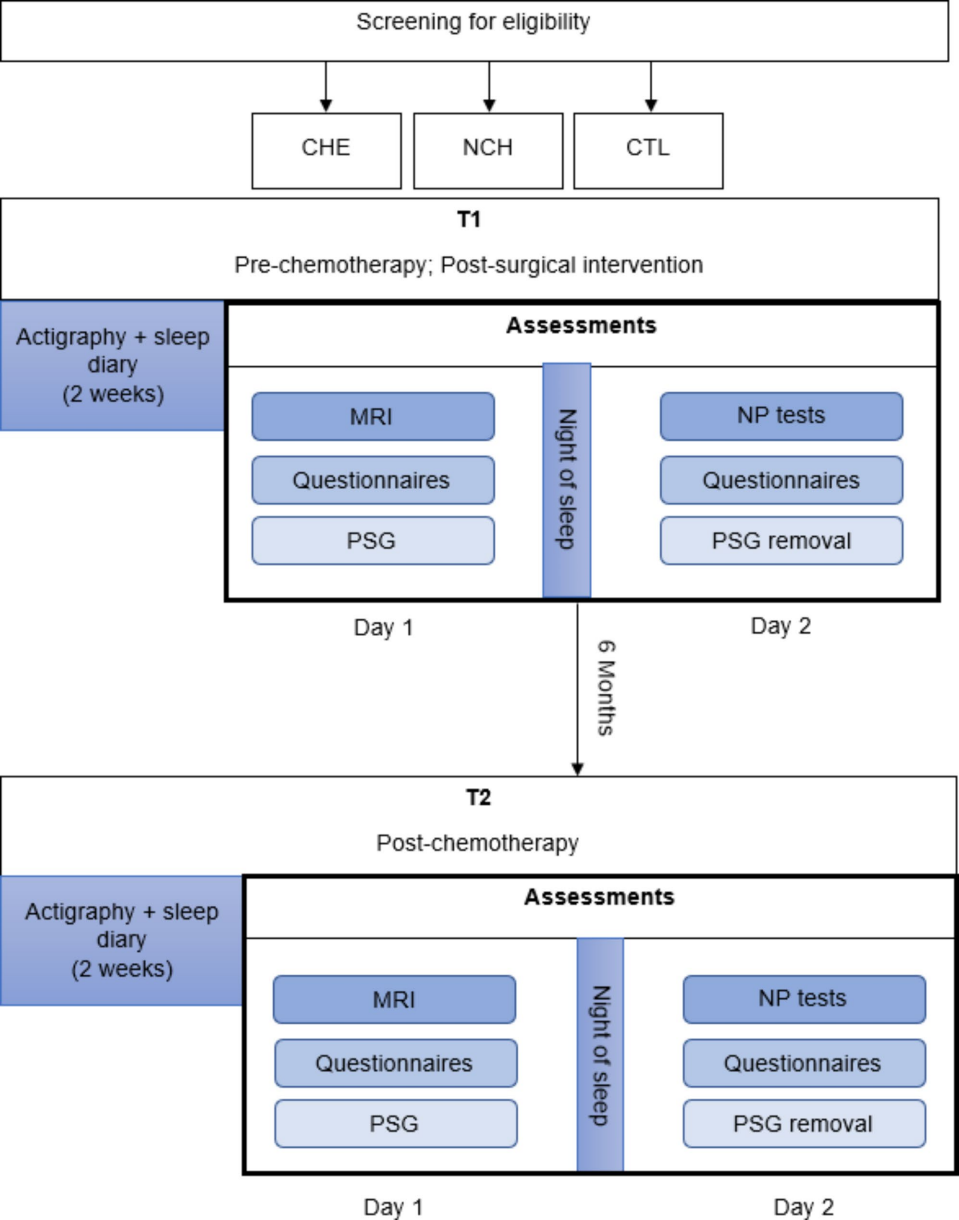
ICANSLEEP-1 trial design. CHE: group of patients treated with chemotherapy; NCH: group of patients not treated with chemotherapy; CTL: control group; MRI: magnetic resonance imaging; PSG: polysomnography; NP tests: neuropsychological tests

The modalities of participation for each of the three groups are summarized in Fig. 1. The overview of study assessments is given in Table 2.

**Table 2:** Questionnaires and neuropsychological test battery included in the ICANSLEEP-1 study with their outcome measures

Domains	Assessment tests	Outcome measures	Range	Evaluated time frame
Sleep Questionnaires				
Insomnia	ISI	Total Score	0–28 (> 7)	Over the last two weeks
Sleep Quality	PSQI	Total score	0–21	Over the last month
Circadian Typology	MEQ	Total score	16–86	-
Quality of Life Questionnaires				
Cognitive complaint	Fact-Cog	PCI	0–72	Over the last seven days
		PCA	0–28	
		QoL	0–16	
		Oth	0–16	
Quality of life	FACT-G (patients)	Total score	0–108	Over the last seven days
Pain	BPI	Severity of pain	0–10	Over the last eight days
		Interference of pain	0–10	
Fatigue	FACIT-F (patients)	Total score	0–52	Over the last seven days
	MFI-20	General and physical fatigue	9–45	-
		Reduced activity	3–15	
		Reduced motivation	2–10	
		Mental fatigue	6–30	
Anxiety	STAI-Y	State score	20–80	At the time being
		Trait score	20–80	Generally speaking,
Depression	BDI-II	Total score	0–63	Over the last two weeks
Neuropsychological tests				
Global functioning	MoCA	Total Score	0–30	
Episodic Memory	HVLT	Total free recall score	0–36	
		Delayed recall score	0–12	
Working Memory	WMS-III subset of spatial memory	Total number of correct trials forward	0–16	
		Total number of correct trials backward	0–16	
	Baddeley dual Task	Digit span: number of correct sequences	3–10	
		Motor Task: number of boxes checked in 2 min	0–332	
		Dual task: number of correct sequences; number of boxes checked	0–332	
Attention	D2-R	Number of target characters processed	0–513	
		E %: [(EO + EC)/PTO] x100	0–100	
Executive Functions	N-Back	Number of correct responses	0–48	
	TMT (A – B)	Time in seconds of part A	≥ 0s	
		Time in seconds part B – A	≥ 0s	
	Stroop	Naming	≥ 0s	
		Reading	≥ 0s	
		Interference Time	≥ 0s	
	Verbal Fluency	Letters P & R		

*ISI*: Insomnia Severity Index, *PSQI*: Pittsburgh Sleep quality Index, *MEQ*: Morningness-Eveningness Questionnaire, *FACT-COG*: Functional Assessment of Cancer Therapy – Cognitive Function, *PCI*: Perceived Cognitive Impairment, *PCA*: Perceived Cognitive Abilities, *QOL*: impact on Quality of Life, *Oth*: Comments from Others, *FACT-G*: The Functional Assessment of Cancer Therapy – General, *BPI*: Brief Pain Inventory, *FACIT-F*: Functional Assessment of Chronic Illness Therapy – Fatigue, *MFI-20*: Multidimensional Fatigue Inventory, *STAI-Y*: State-Trait Anxiety Inventory, *BDI-II*: Beck’s Depression Inventory-2nd edition, *MoCA*: Montreal Cognitive Assessment; *HVLT*: Hopkins Verbal Learning Test; *WMS-III*: Wechsler Memory Scale – 3rd edition, *D2-R*: d2 Test of Attention-Revised, *E%*: percent of Errors, *EO*: Errors of Omission, *EC*: Errors of Commission, *PTO*: Processed Target Objects, *TMT*: Trail Making Test

Once signed consent is obtained, the inclusion evaluation will aim to assess sleep complaints and sleep apnea. After inclusion, patients from the CHE group will be evaluated in two sessions spaced 6 months apart: before the first administration of adjuvant chemotherapy (baseline or T1) and 2 weeks after the end of adjuvant chemotherapy (T2). The same will apply to patients in the NCH group and healthy controls.

Each session will be conducted over two half-days separated by a night of sleep. On the afternoon of day 1, structural MRI assessments will be realized, and the participants will be asked to complete self-report questionnaires related to psychological factors, after which PSG will be placed to measure sleep patterns during one full night of sleep at home. Self-report questionnaires on quality of sleep and quality of life will be filled in by participants at home. On the morning of day 2, PSG will be removed, and the participants will undergo neuropsychological tests and fill in the other questionnaires.

### Assessments tools

An overview of tools to be used in the study is given in Table 2.

### Self-reported questionnaires

The *Insomnia Severity Index* (ISI) [54] is a self-report questionnaire assessing the nature, severity, and impact of insomnia. It evaluates the following dimensions: severity of sleep onset, sleep maintenance, early morning awakening problems, sleep dissatisfaction, interference of sleep difficulties with daytime functioning, noticeability of sleep problems by others, and distress caused by the sleep difficulties. A score ≥ 7 will be considered to indicate the presence of insomnia.

The *Berlin questionnaire* [55] will be used to assess sleep complaints and sleep apnea factors such as snoring behavior, waketime sleepiness or fatigue, and the presence of obesity or hypertension.

The *Pittsburgh Sleep Quality Index* (PSQI) [56] is a self-report questionnaire that will be used to assess efficiency and quality of sleep over a 1-month time interval. It measures the following components: subjective sleep quality, sleep latency, sleep duration, habitual sleep efficiency, sleep disturbances, use of sleeping medication, and daytime dysfunction. A score > 5 is considered to indicate a significant sleep disturbance.

The *Morningness-Eveningness Questionnaire* [57] will be used to assess chronotype given that it can influence the ambulatory measurement of rest-activity rhythm in healthy volunteers [58]. The questionnaire contains 19 questions to assess morningness-eveningness. At the end, the sum of the scores will be converted to a 5-point morningness-eveningness scale, with a score of 70–86 meaning that the person is “definitely morning type,” a score of 59–69 meaning that the person is “moderately morning type,” a score of 42–58 meaning that the person is “neither type,” a score of 31–41 meaning “moderately morning type,” and a score of 16–30 meaning “definitely evening type.” The *Functional Assessment of Cancer Therapy Cognitive Scale* (FACT-Cog) [59] in its French version will be used to assess subjective cognitive complaints and their impact on the quality of life. Memory, attention, concentration, language, and thinking abilities will be assessed with 37 items on a 5-point Likert scale (from 0 = not at all to 4 = very much).

The *Functional Assessment of Cancer Therapy – General* (FACT-G) [60] will assess general quality of life using 27 items categorized under the following components: physical, social/family, emotional, and functional well-being. The score range is 0–108, noted on a 5-point Likert scale (from 0 = not at all to 4 = very much).

The *Brief Pain Inventory* (BPI) [61] is a self-assessment pain questionnaire. It evaluates pain intensity on a sensory dimension and the level of interference of pain in the participant’s life. It assesses the following components: pain relief, pain quality, and patient’s perception of the cause of pain. A score of 1–4 = mild pain, 5–6 = moderate pain, and 7–10 = severe pain.

Fatigue will be assessed using the *Functional Assessment of Chronic Illness Therapy* (FACIT-F) [62] and the *Multidimensional Fatigue Inventory* (MFI-20) [63]. The first is used in its short form as a 13-item self-report questionnaire that aims to assess participants’ perception of fatigue and how it might affect their daily activities and functioning. All items are noted on a 5-point Likert scale (from 0 = not at all to 4 = very much). The second is also a self-report questionnaire designed to measure five fatigue components (general fatigue, physical fatigue, reduced activity, reduced motivation, and mental fatigue). Each question is noted from 1 = no, it is not true to 5 = yes, it is true. The score ranges from 4 to 20. A higher score indicates greater fatigue. The specificity of this questionnaires is that it can be administered to both healthy controls and BC patients. This will allow us to compare both groups.

The *State-Trait Anxiety Inventory for Adults* (STAI-Y) [64] will be used to evaluate anxiety. It contains two separate scales to measure the state (where participants describe how they feel in the moment) and the trait (where participants describe how they generally feel) of anxiety. Each scale contains 20 statements. To measure the state of anxiety, the 20 statements evaluate how the subject feels “right now,” while the trait is measured by asking the subject how they feel “generally”. The statements are scored on a 4-point Likert scale from 1 = no to 4 = yes for the state component and from 1 = never to 4 = almost always. The total score for both scales ranges from 20 to 80, with higher scores indicating more severe anxiety.

The *Beck’s Depression Inventory* (BDI-II) [65] will be used to evaluate depression using a 21-item scale that provides information about the severity and nature of depression in the participants. The items range from 0 = the absence of symptoms to 3 = an intense level. The sum of the score will define the presence and severity of depression. A score of 1–10 = absence of depression, 11–16 = mild mood disturbance, 17–20 = borderline clinical depression, 21–30 = moderate depression, 31–40 = severe depression, and finally, a score over 40 = extreme depression.

#### Sleep and circadian rhythm assessments

##### Salivary cortisol to assess diurnal cortisol rhythm

Saliva collection will be performed to measure levels of salivary cortisol, one of the markers of circadian rhythms. The procedure involves inserting a cotton ball into the mouth and soaking it with saliva for 2–3 min, then putting it back into the storage tube. Participants will be invited to perform these saliva extractions at home as follows: before sleep, at awakening, and 30 and 45 min after awakening.

##### Actigraphy to assess rest-activity rhythm

Two weeks before each assessment time point, participants will place an actigraph MotionWatch® 8, (CamNtech Ltd, UK) on their non-dominant wrist (Fig. 1). Actigraphy will provide information related to parametric and non-parametric measures of rest-activity rhythm, as previously published in BC patients [29, 66, 67]. The actigraphs contain a triaxial sensor detecting acceleration in a 0.01–8 g range. They will set to 30-second epochs to allow sleep parameter analysis (validated using 30-second epochs by the manufacturer) and record light intensity to aid the analysis of sleep episodes. During these 2 weeks, participants will also be asked to fill in a sleep diary that will be used to analyze actigraph data for better accuracy. Participants will subjectively indicate their sleep quality and duration, including hours in bed, sleep quality, number and duration of nocturnal awakenings, and naps and their duration. Moreover, the actigraphs allow the tracking of both the diurnal physical activity and the nighttime activity of participants, given that physical activity could also influence sleep in BC patients and being active or not could influence the results [68, 69].

##### Polysomnography to assess sleep structure

During the night, sleep will be recorded using ambulatory PSG (Siesta, Compumedics) at home or at the hotel where some participants will stay. Recordings of brain activity using electroencephalography (EEG), eye movements, heart rate, respiratory rate, and oxygen saturation will be performed simultaneously. These measures will offer us quantitative data on sleep onset latency, sleep efficiency, number of nocturnal awakenings after sleep onset, total sleep time, and the relative percentages of sleep stages. Twenty EEG electrodes will be placed on the scalp, over prefrontal (FP1/FP2), frontal (F3/F4/F7/F8/Fz), central (C3/C4/Cz), temporal (T3/T4), parietal (P3/P4/Pz), and occipital (O1/O2) sites, according to the international 10–20 system, using Ag/Au electrodes with a ground and a bi-mastoids reference. The impedance for all electrodes will be kept below 5 kΩ. The hardware EEG filter band pass will be 0.15–121 Hz and the sample rate will be 256 Hz. Two electrodes will be placed above and below the eyes to record eye movements, along with two electrodes on the chin to measure muscle tone. An electrocardiogram will also be recorded by placing two electrodes under each clavicle. To detect potential sleep apneas or hypopneas, thoracic and abdominal belts will be placed to record respiratory movements, a microphone to detect snoring, nasal pressure, nasal and oral thermistors to measure respiratory airflow, and a finger pulse oximeter to measure oxygen saturation. In addition, analyses of spindles and slow waves will be performed using the open-source SpiSOP (www.spisop.org; RRID: SCR_015673) based on MATLAB 2017b (MathWorks, Natick, USA; RRID: SCR_001622). For this work, the standard settings of SpiSOP will be used based on previously published algorithms [70]; SpiSOP documentation is available here: www.spisop.org/documentation/.

#### Neuropsychological test Battery

Global cognitive functioning will be assessed with the Montreal Cognitive Assessment (MoCA) [71] at baseline only (T1). During each session, cognition will be assessed for all participants using standardized neuropsychological tests, all while accounting for the test-retest effect (Table 2). Episodic memory will be assessed using the Hopkins Verbal Learning Test (HVLT) [72], standardized and adapted for French populations. Working memory will be assessed using the subtest of Spatial Memory forward and backward (Wechsler Memory Scale-III) and by the dual task of Baddeley [73]. Attention will be assessed using d2-R [74]. Finally, executive function will be assessed by the Stroop test [75], the Trail Making Test (B-A) [64], lexical fluency (letters P & R) [76], and by a modified version of the N-Back task specifically developed for the AGING protocol [77]. Sleep-dependent memory consolidation will be evaluated using a computer-based memory task performed both before and after sleep (i.e., learning in the afternoon and recall the next morning) in order to measure associated grapho-elements [78].

#### Brain structural magnetic resonance imaging assessments

Structural MRI will be obtained using a 3T GE (Signa Premier). The whole procedure will be described to all participants before beginning the acquisitions. Volumetric T1-weighted images will be acquired at a 1 mm^3^ isotropic resolution to measure grey matter density and volume using a three-dimensional (3D) fast field echo sequence (sagittal acquisition; repetition time, 2.2 s; echo time, 2.7 ms; flip angle, 8°; 180 slices; slice thickness, 1 mm; matrix size, 256 × 256). Then, diffusion tensor imaging (DTI) (2D) will be used to measure white matter microstructural integrity (128 directions, multi-shell; repetition time, 4.8 s; echo time, 71 ms; flip angle, 90°; slice thickness, 2 mm; matrix size, 108 × 108). Moreover, a T2-FLAIR (2D) will be acquired to make sure that the participants do not have any brain lesions (axial; repetition time, 8.5 s; echo time, 90 ms; inversion time, 2.4 s; flip angle, 160°; slice thickness, 4 mm).

## Statistical considerations

This study was designed to control an error risk α of 0.05 and a power of 80%. Assuming sleep alterations for 30% of patients following chemotherapy compared to a theoretical proportion of 10% [31], the required sample size is 24 patients per group. We thus planned to enroll a total of 75 participants (25 BC patients receiving chemotherapy, 25 BC patients without an indication for chemotherapy, and 25 healthy women).

Statistical analyses will be performed using R® software. Exploratory data analyses will provide, for qualitative variables, the frequencies and their exact 95% confidence intervals, and for quantitative variables, the mean, the standard deviation of the mean, the median, and the quartiles.

Repeated measures analyses of variance (ANOVA) will be applied to longitudinally compare BC patients and healthy controls, with p < 0.05. The relationship between sleep parameters and scores on cognitive tests and quality of life assessments will be assessed at any time using a linear mixed model (accounting for the correlation between repeated measures of the same subject). SPM12 software (https://www.fil.ion.ucl.ac.uk/spm/software/spm12) will be used to assure a whole-brain voxel-based analysis and FSL 6.0.5 software will be used to preprocess white matter data (https://fsl.fmrib.ox.ac.uk/fsl/fslwiki). The relationships between white matter integrity, sleep parameters, and scores on cognitive tests will be assessed at any time using a linear mixed model (considering the correlation between repeated measures of the same subject). Similarly, relationships between sleep disturbances and circadian disruptions will be assessed at any time using a linear mixed model.

## Data management

A web-based data capture (WBDC) system will be used for data collection and query handling. The investigator will ensure that data are recorded on the eCRFs as specified in the study protocol and in accordance with the instructions provided.

The investigator ensures the accuracy, completeness, and timeliness of the data recorded and of the provision of answers to data queries according to the Clinical Study Agreement. The investigator will sign the completed eCRFs. A copy of the completed eCRFs will be archived at the study site [79].

## Withdrawal from study

The reasons a participant may discontinue participation in the study include the following circumstances:

- Intercurrent event, not compatible with the pursuit of the study.
- Woman’s decision (the data already collected during the search can be kept and exploited unless she opposes it).
- Participant lost of view.
- Investigator’s decision.

## Discussion

Recent research in BC patients has associated the presence of cognitive deficits with the occurrence of sleep complaints [80–82] but the findings remain inconclusive [14] and further investigations on this topic are needed.

Although sleep disturbances are thought to be a side effect of cancer treatment, notably chemotherapy [13, 83], multiple studies showed that sleep complaints in BC exist even in BC patients who did not receive chemotherapy [84, 85] and their presence has detrimental effects on patients’ quality of life [86]. Sleep is intrinsically linked to circadian rhythms and a recent review highlighted the presence of circadian disruptions in BC, more specifically of cortisol and rest-activity rhythms [13]. These disruptions are, in most cases, associated with chemotherapy in this disease but were also found outside chemotherapy [67] and may contribute to the development and persistence of sleep disturbances—among other symptoms—leading many women with BC to complain about their quality of sleep [83, 87].

Longitudinal studies comparing sleep disturbances in BC patients before and after receiving chemotherapy are thus needed to better understand their influence on cognitive functioning in this pathology. Thus, our study is designed to assess sleep before and after adjuvant chemotherapy using PSG in BC patients treated or not with adjuvant chemotherapy compared to healthy controls. The use of PSG will bring us deeper knowledge of sleep structures (i.e., sleep stages, quality, and quantity) as well as memory consolidation processes during sleep using EEG-derived indices. In addition, PSG will allow us to assess sleep apnea in all participants [45]. Moreover, using DTI, we will be able to provide information related to white matter integrity and its association with both sleep disturbances and memory deficits [88–90].

Accordingly, the characterization of sleep disturbances as contributors to cognitive decline in BC will increase awareness among clinicians to focus on these underdiagnosed symptoms, leading to the development of more appropriate interventions. Improving sleep in BC will lead to the enhancement of patients’ cognitive functioning and possibly their daily lives. A better understanding of sleep disturbances in BC will pave the way for future studies dedicated to improving both sleep and cognitive functioning in BC patients. For now, previous reports have shown the beneficial effects of cognitive behavioral therapy and physical activity on sleep complaints, self-esteem, and quality of life [69, 91].

This study has some limitations. For example, participants may be uncomfortable in the MRI machine due to the narrow space and acoustic noise, which will be reduced using methods such as the passive absorption of acoustic noise by earplugs and helmets. Time will also be dedicated to installing participants comfortably and explaining the MRI protocol to reduce stress in the machine. PSG installation could also bother participants. However, our experience has shown that participants accept PSG well, especially because it will be implemented in ambulatory form at the participant’s home for most participants. Measuring sleep in a familiar environment can help reduce the bother and anxiety caused by PSG examination, as well as the first-night effect.

## Conclusion

Using a longitudinal approach, the ICANSLEEP-1 study is expected to provide information on sleep structure modifications in BC patients both before and after chemotherapy. Thanks to the objective evaluation of sleep together with measures of two circadian rhythms, associated with a neuropsychological test battery and structural MRI acquisition, this study is intended to generate advanced knowledge on the altered sleep processes in BC and their impact on cognition and cerebral structures. In the long term, we expect that our results will be useful to both patients and clinicians, allowing a better understanding of patient complaints and thus improving their supportive care and quality of life.

## Data Availability

Not applicable.

## Abbreviations

BC: breast cancer
PSG: polysomnography
EEG: electroencephalography
MRI: magnetic resonance imaging
DTI: diffusion tensor imaging
CNS: central nervous system
HPA: hypothalamic-pituitary-adrenal
